# Efficacy and safety of the Chinese herbal medicine Bu-Shen-Huo-Xue granule for the treatment of coronary heart disease: study protocol for a multicenter, randomized, double-blinded, placebo-controlled clinical trial

**DOI:** 10.3389/fcvm.2024.1293818

**Published:** 2024-06-04

**Authors:** Lanchun Liu, Jie Wang, Jun Li, Xiao Li, Rong Li, Yongmei Liu, Guang Yang, Qiyuan Mao, Lin Wang, Zhengyang Yao, Yongcheng Wang, Shuli Zong, Chao Liu

**Affiliations:** ^1^ Department of Cardiology, Guang’anmen Hospital of China Academy of Chinese Medical Sciences, Beijing, China; ^2^ Department of Cardiology, Affiliated Hospital of Shandong University of Chinese Medicine, Jinan, Shandong, China; ^3^ Department of Cardiology, First Affiliated Hospital of Guangzhou University of Chinese Medicine, Guangzhou, Guangdong, China

**Keywords:** coronary heart disease, kidney deficiency, blood stasis, Bu-Shen-Huo-Xue, Chinese herbal medicine, traditional Chinese medicine, randomized controlled trial

## Abstract

**Background:**

Coronary heart disease (CHD) is representative of cardiovascular disease and the leading cause of death in humans. Previous studies have shown that kidney disease is associated with CHD, and current treatment options that can improve both cardiac and renal functions still have some limitations. The traditional Chinese medicine Bu-Shen-Huo-Xue granule (BSHXG) can promote blood rheology, inhibit platelet agglutination, and improve heart and kidney functions.

**Methods:**

This is a multicenter, randomized, double-blind, placebo-controlled clinical trial. A total of 210 participants will be randomized to the intervention group and the placebo group. The Guang’anmen Hospital of China Academy of Chinese Medical Sciences is the leading center, and the Affiliated Hospital of Shandong University of Chinese Medicine and the First Affiliated Hospital of Guangzhou University of Chinese Medicine are the participating units. In addition to conventional pharmacotherapy for angina, the intervention group will receive BSHXG while the placebo group will receive BSHXG placebo. All participants will receive 2 months of treatment with 6 months of follow-up. The primary outcome is the efficacy of angina pectoris symptoms in CHD. Secondary outcomes are nitroglycerin arrest, ECG efficacy, Seattle Angina Questionnaire score, serology indicators, assessment of safety, and cardiovascular endpoint events. The transcriptome and metabolome will be used to screen biomarkers for diagnosis and efficacy evaluation.

**Discussion:**

This study aimed to evaluate the efficacy and safety of Bu-Shen-Huo-Xue granule in the treatment of coronary heart disease, and to evaluate the benefits to patients with coronary heart disease from both cardiac and renal indicators.

**Trial registration:**

This trial is approved by the Ethical Review Committee of the Guang’anmen Hospital China Academy of Chinese Medical Sciences with the number 2022-224-KY-01, and has been registered on the Chinese Clinical Trials Registry with the number ChiCTR2300070977 on 27 April 2023.

## Background

Cardiovascular-related diseases, as a major public health problem, were noted in the World Health Organization (WHO) Global Health Estimate Report 2022 (1), which states that about 33.2 million deaths occur worldwide from cancer, cardiovascular disease, diabetes, and chronic respiratory diseases due to increased population growth and increased life span. The “Summary of China Cardiovascular Health and Disease Report 2021” (2) also points out that the prevalence and mortality of cardiovascular diseases in China is still on the rise, with rural and urban cardiovascular diseases accounting for 46.74% and 44.26% of the causes of death, respectively, resulting in an increasing burden. Coronary heart disease (CHD) with unstable angina pectoris is a condition where the arteries very easily rupture and bleed because of plaques, and the prognosis is often dangerous. Although the existing secondary prevention concepts and treatments such as interventional therapy and coronary artery bypass grafting have achieved effective results, some patients still developed recurrent angina pectoris after revascularization, following a decline in quality of life, resistance against antiplatelet drugs, secondary formation of thrombosis, and concurrent anxiety and depression. These remain the clinical challenges that need to be addressed. A growing number of epidemiological studies have reported a link between chronic kidney disease (CKD) and CHD, perhaps providing another perspective on clinical treatment. Traditional Chinese Medicine (TCM) has been widely used in treating CHD for a long time and studies have confirmed the biological mechanism (3, 4). With the change of social lifestyle, the TCM syndrome for unstable angina pectoris with coronary heart disease presents a combination form of “kidney deficiency and blood stasis.” A clinical epidemiological survey on the distribution of TCM syndromes showed that qi deficiency was the main symptom of angina pectoris in coronary heart disease, accounting for up to 78.8%, second only to blood stasis, of which heart and kidney qi deficiency was the mainstay (5). Another study analyzed nine common symptom combinations of patients with angina pectoris through algorithms, of which the symptom group with qi deficiency and blood stasis as the main evidence accounted for as high as 21% (6). Studies have described the correlation between estrogen level and renal deficiency-type coronary heart disease and the intervention of kidney tonifying Chinese medicines (7). Bu-Shen-Huo-Xue Granule (BSHXG) is a common Chinese medicine for prevention and cure of CHD. It is based on the traditional Chinese medicine theory of “Tonifying the kidney and promoting blood circulation”. The existing research has some problems such as insufficient sample size, low quality, and insufficient evidence, therefore, we designed a multicenter, randomized, double-blind, placebo-controlled, clinical trial to evaluate the efficacy and safety of BSHXG in the treatment of CHD.

## Objective

To evaluate the efficacy and safety of BSHXG in the treatment of kidney deficiency and blood stasis in unstable angina pectoris coronary heart disease. The mechanism of the drug will be explored from the transcriptomics and metabolomics levels, which provide a basis for the precise treatment of diseases.

## Methods/design

### Study design

**Figure 1:**
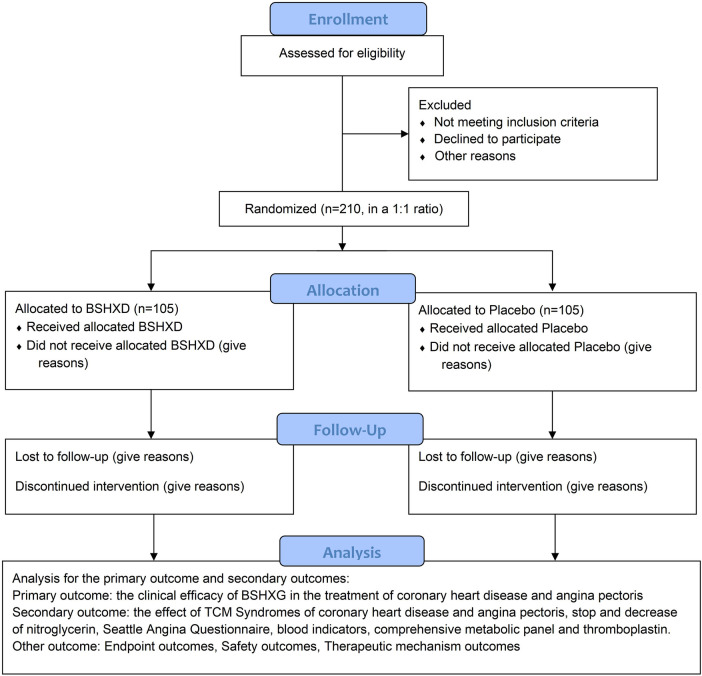
The flow chart of the trial.

This study is a multicenter, randomized, double-blind, placebo-controlled trial registered with the Chinese Clinical Trials Registry (registration number: ChiCTR2300070977). All documents, including study protocols, informed consent forms, and case report forms (CRFs) meet the requirements of the Declaration of Helsinki and have been reviewed by the Academic Committee and Ethics Committee of the Guang’anmen Hospital. The trial will be implemented based on the principles of Good Clinical Practice and reported according to the CONSORT statement (8). The trial flow diagram is illustrated in Figure 1.

### Participants

#### Sample size

This study was a randomized controlled trial, and the frequency of weekly angina attacks in participants was the primary outcome measure. One previous study suggested that the reduction of weekly angina attacks after interventional treatment is 4.2 with a standard deviation of 8.5 (9). Therefore, in the following formula, *c* is the ratio between two sample cases, *n*1 = *n*2, so *c* = 1. The variable σ is the standard deviation of 8.5, and *δ* is the expected effect of 4.2, so *σ* = 8.5 and *δ* = 4.2. Given a type I error rate of *α* = 0.05, and a power of 90% (type II error rate of *β* = 0.1), then *uα* = 1.96, *uβ* = 1.282. *n*1 = *n*2 = 88, the sample size for one group needs to be 88, resulting in *n* = 2 × 88 = 176 patients. Considering the maximum possible dropout rate is 15%, a total of 210 patients were needed to be allocated to reach the required number of patients for the efficacy analysis.

$$n={\left[\frac{(u\alpha +u\beta )\times \sigma }{\delta }\right]}^{2}\frac{\left(1+c\right)}{c},\;n2=cn1$$

#### Inclusion criteria

1. Aged 40–80 years old, male or female.
2. Typical chest pain behind the sternum, predictably accompanied by pressure, burning, and dying sensations.
3. Induced or aggravated by fatigue, emotional excitement, heavy meals, or cold.
4. Chest pain symptoms lasting for 3–5 min each time and lasting less than 30 min.
5. Can be relieved by rest and/or nitroglycerin within minutes.
6. Canadian Cardiovascular Society (CCS) Angina Grade II–III.
7. Must have experienced symptoms for a minimum specified period (at least 3 months) to confirm the instability of the angina. And there has been no worsening of the above symptoms in the past 3 months.
8. Patients who were diagnosed with kidney deficiency and blood stasis syndrome by TCM syndromes.
9. Provide previous medical records and examination reports that have a clear history of myocardial infarction, or coronary artery bypass grafting. Coronary angiography shows diameter stenosis of one or more main coronary arteries >70%, or diameter stenosis of the left main coronary artery >50%.
10. Patients who voluntarily sign the informed consent.

#### Exclusion criteria

1. Severe valvular heart disease, severe neurosis, menopausal syndrome, hyperthyroidism, cervical spondylosis, biliary heart disease, stomach and esophageal reflux, and other non-coronary heart disease caused by chest pain.
2. Poor hypertension control (under drug control, blood pressure ≥160/100 mmHg at rest and occasionally within a week), severe cardiopulmonary insufficiency [ejection fraction (EF) <35%], severe arrhythmia (rapid atrial fibrillation, atrial flutter, paroxysmal ventricular tachycardia, atrioventricular block of type II and above, complete bundle branch block).
3. Complicated with serious primary diseases such as heart, brain, liver, kidney, hematopoietic system [hepatic function alanine aminotransferase (ALT) or aspartate transaminase (AST) value >1.5 times the upper limit of normal, patients with abnormal renal function], insulin-dependent type 2 diabetes.
4. Patients with depression or anxiety.
5. Pregnant or lactating women.
6. Patients with malignant tumors.
7. Patients with allergies and allergies to test drug components.
8. Patients with poor compliance and low possibility of follow-up.
9. Participants in other clinical trials within the past month.
10. Other major diseases or factors that interfere with the completion of the trial or affect the interpretation of the results.

#### Randomization

The parallel randomization method was adopted, and the center was stratified and divided into experimental group and placebo group according to the ratio of 1:1. With the help of the SAS statistical software PROC PLAN process statement, the treatment allocations with serial numbers 001–210 were listed.

#### Blinding

1. Drug packaging and distribution: according to the standardized operation steps of double-blind clinical trials, the experimental drug and placebo drug are packaged and distributed.
2. Blind code preservation regulations: adopt double-blind design in duplicate, separately sealed, and respectively stored.
3. Unblinding regulations: after all the research data are entered and locked, the staff of Guang’anmen Hospital are unblinded, and the statistical analyst writes a statistical analysis report.
4. Regulations on blinding individual cases in emergencies: when serious adverse events occur or emergency rescue is required, the main investigator decides whether it is necessary to open the emergency letter.
5. Provisions for the failure of double-blind test: blind leakage or emergency letter reading rate exceeds 20%.

#### Withdrawal criteria and management

1. Patients who do not meet the inclusion criteria.
2. Patients who have never used drugs after inclusion.
3. Patients with serious safety problems during the trial.
4. Patients whose drug treatment is ineffective and has no effect or clinical value.
5. Patients with non-fatal myocardial infarction, revascularization, and sudden cardiac death during the trial.

#### Intervention

BSHXG consists of 10 kinds of Chinese Herbs (Table 1), including *Salvia miltiorrhiza* (Dan Shen), *Ligusticum wallichii* (Chuan Xiong), *Peaoniae radix rubra* (Chi Shao), *Morinda officinalis* (Ba Ji Tian), *Fructus lycii* (Gou Qi Zi), *Cistanches herba* (Rou Cong Rong), *Fructus trichosanthes* (Gua Lou), *Ginseng radix et rhizoma* (Ren Shen), *Cassia twig* (Gui Zhi), and *Panax notoginseng* (San Qi). It has a variety of pharmacological effects, including antioxidation, antiatherosclerosis, lipid-lowering, and antiplatelet aggregation.

**Table 1:** The action of each traditional Chinese medicinal herb.

Chinese name	English name	Origin	Weight (%)^a^
Chuan Xiong	*Ligusticum wallichii*	The dry rhizome of *Ligusticum*	11.65
Dan Shen	*Salvia miltiorrhiza*	The dried roots and rhizomes of *Salvia miltiorrhiza* Bge	11.08
Chi Shao	*Peaoniae radix rubra*	The dried root of *Paeonia lactiflora* Pall	8.88
Rou Cong Rong	*Cistanches herba*	The dried scaled fleshy stalk of *Cistanche deserticola* Y.C. Ma	7.42
Ba Ji Tian	*Morinda officinalis*	The dry roots of *Morinda officinalis* How	11.65
Gua Lou	*Fructus trichosanthes*	Dry ripe fruit of *Trichosanthes rosthornii* Harms	12.22
Gui Zhi	*Cassia twig*	The dry shoots of *Cinnamomum cassia* Presl	3.91
San Qi	*Panax notoginseng*	The dried root or rhizome of *Panax notoginseng* F.H. Chen	12.22
Ren Shen	*Ginseng radix et rhizoma*	The dried roots and rhizomes of *Panax ginseng* C. A. Mey	7.01
Gou Qi Zi	*Fructus lycii*	The dried ripe fruit of *Lycium barbarum*	13.94

^a^ The weight of every single herb in each bag of BSHXG (12.27 g).

#### Standard therapy

Western medicines for the treatment of coronary heart disease are allowed to be used in accordance with guidelines and clinically reasonable standards. Drugs that relieve symptoms and improve ischemia mainly include three categories, namely, β-blockers, nitrate drugs, and calcium channel blockers. In addition, trimetazidine can improve myocardial tolerance to ischemia and left ventricular function, as well as relieve angina pectoris, which can be used as a second-line drug by regulating myocardial energy substrates and increasing the aerobic oxidation ratio of glucose. When beta-blockers are contraindicated, ineffective, or have adverse reactions, nicorandil and ivabradine can be used to relieve symptoms. Drugs can be used to improve the prognosis and prevent adverse cardiovascular events such as myocardial infarction and death, mainly including antiplatelet drugs, lipid-lowering drugs, angiotensin-converting enzyme inhibitors (ACEI), or angiotensin II receptor antagonists agent (ARB). Detailed medication and dosage recommendations are provided in Table 2.

**Table 2:** Total daily dose of antianginal medication.

Medication	Total daily dose in mg
Drugs that relieve symptoms and improve ischemia	
Bisoprolol	5
Metoprolol tartrate	25
Amlodipine	2.5
Nifedipine	20
Isosorbide mononitrate	30
Trimetazidine	20
Nicorandil	20
Ivabradine	5
Drugs that improve prognosis	
Aspirin	75
Tigrillo	120
Ezetimibe	10
ACEI/ARB	10

#### Experimental group

Drug name: Bu-Shen-Huo-Xue formula; Dosage form: granules; Specification: g/bag; Production unit: provided by Sichuan New Green Pharmaceutical Technology Development Co., Ltd.; Storage conditions: dry and ventilated place. Medication method of the experimental group: On the basis of conventional Western drug treatment, one bag of BSHXG is taken with warm boiled water at 0.5 h interval after intake of Western medicine. It is orally administered twice in the morning and evening, and the course of treatment is 8 weeks.

#### Control group

Placebo name: Bu-Shen-Huo-Xue placebo formula; Dosage form: granules; Specification: g/bag; Production unit: provided by Sichuan New Green Pharmaceutical Technology Development Co., Ltd.; Storage conditions: dry and ventilated place. Medication method of the control group is the same as for the experimental group.

#### Outcome measurement

Changes in the following outcomes from baseline (week 0) to intervention endpoints (week 8) will be assessed.

#### Primary outcome

The primary outcome is to validate the clinical efficacy of BSHXG in the treatment of coronary heart disease and angina pectoris, including the values of change in the frequency and duration of angina before and after treatment.

(1) Effective: angina pectoris symptoms disappear or basically disappear.
(2) Improved: The frequency of angina pectoris attacks is reduced and the duration is shortened although it still exists.
(3) Ineffective: The frequency and duration of angina pectoris attacks are basically the same as before treatment.
(4) Aggravated: The frequency and duration of angina pectoris attacks increase.

#### Secondary outcomes

1. The effect of TCM syndromes of coronary heart disease and angina pectoris
  (1) Effective: TCM syndrome improvement >70%.
  (2) Improved: TCM syndrome improvement >30%.
  (3) Ineffective: TCM syndrome improvement <30%.
2. Stopping and decreasing of nitroglycerin
  (1) Effective: Angina pectoris disappears or is basically relieved within 3 min (including 3 min) after taking the medicine.
  (2) Improved: angina pectoris disappears or is basically relieved within 3–5 min after taking the medicine.
  (3) Ineffective: Angina pectoris gradually relieves or does not improve more than 5 min after taking the medicine.
  (4) Aggravated: Angina pectoris worsens after taking medication.
3. Seattle angina questionnaire
  (1) Effective: standard points increase.
  (2) Ineffective: the standard integral remains unchanged.
  (3) Aggravated: reduction of standard points.
4. Blood indicators such as routine blood test, comprehensive metabolic panel, and thromboplastin
  (1) Effective: The difference (mean ± standard deviation) between post-treatment and baseline is statistically significant (*p* < 0.01).
  (2) Improved: The difference (mean ± standard deviation) between post-treatment and baseline is significant (*p* < 0.05).
  (3) Ineffective: The difference (mean ± standard deviation) between post-treatment and baseline has no significance (*p* > 0.05).
  (4) Aggravated: The difference between post-treatment and baseline (mean ± standard deviation) is opposite to the outcome of the treatment effect.

#### Endpoint outcomes

Cardiovascular endpoint events including all-cause mortality, non-fatal myocardial infarction, stroke, percutaneous coronary intervention (PCI) surgery, coronary bypass surgery, malignant arrhythmias, worsening angina pectoris, and re-hospitalization were followed 6 months after treatment. If the patient experiences any discomfort, new changes, or unexpected conditions during the trial research period, whether related to the intervention or not, he or she will be clinically evaluated and given coronary angiography examination or medical treatment.

1. Valid: no endpoint event occurred.
2. Invalid: an endpoint event occurs.

#### Safety outcomes

1. Vital signs: such as body temperature, blood pressure, breathing, and heart rate (recorded once at each observation point).
2. Routine laboratory tests for blood, urine, and stool (before and after medication).
3. Electrocardiogram (ECG), liver function (ALT, AST), kidney function [blood urea nitrogen (BUN), Creatinine (Cr)] (before and after medication).
4. Adverse events (detailed records at any time).

#### Therapeutic mechanism outcomes

To explore the mechanism of BSHXG in transcriptomics in coronary heart disease renal deficiency blood stasis, the participants are made to give 4 ml of venous blood, which is placed in an anticoagulant separator vacuum collection tube [ethylenediaminetetraacetic acid (EDTA)], stored at 4°C, and peripheral blood nuclear cells (PBNCs) are isolated for no more than 6 h. RNA extraction of peripheral blood nucleated cells was performed in the cardiovascular laboratory to screen for differential molecules in transcriptomics before and after treatment.

#### Data management

1. In the process of this research, the main investigator will appoint a clinical supervisor to regularly conduct on-site supervision visits to the research unit to ensure that all the contents of the research plan are strictly observed, and the information filled in is correct.
2. Personnel participating in clinical trials must carefully study and discuss clinical trial protocols and manuals, and unify recording methods and judgment standards.
3. Researchers should truthfully, in detail, and carefully use pen or carbon pen to fill in the CRF item by item.
4. All observations and findings in the clinical trials should be verified to ensure the reliability of data and that the conclusions in the clinical trials are derived from original data.
5. Medical records and medical record forms are original records and generally cannot be changed. Any corrections may not alter the original records and may only be accompanied by a narrative of justification, signed and dated by the physician participating in the clinical trial.
6. Researchers should actively take measures (notification of follow-up and follow-up) to control the dropout rate of cases within 20%.
7. In order to ensure the compliance of the participants, the participants should fully understand the significance of taking drugs on time. Researchers should keep a record of their medication. Those who do not fully comply with the requirements should be persuaded in a timely manner, and the reasons should be recorded in detail.
8. Each research unit strictly grasps the quality control standards.
9. The accuracy, reliability, and abnormal judgment standards of laboratory tests should be unified among all centers.
10. Laboratory data should be recorded in the CRF and the original report should be glued to the medical record. Laboratory data within the normal range should also be recorded. All researchers involved in data entry and data management will sign a confidentiality agreement to prevent data leakage. The schedule of enrollment, interventions, and assessments can be checked in Table 3.

**Table 3:** Schedule of the data collection.

Process item	Run-in period	Run-in period	Pre-treatment	Treatment period		Follow-up period
Time point	−7 days	−1 day	0 day	4 weeks	8 weeks	6 months
Baseline information						
Informed consent	√					
Eligibility screen			√			
Medical history	√					
Allocation	√					
Effectiveness observation						
Efficacy of angina pectoris			√	√	√	
Efficacy of TCM syndromes			√	√	√	
Stopping and decreasing of nitroglycerin			√	√	√	
Seattle angina questionnaire			√	√	√	
Blood indicators			√		√	
Therapeutic mechanism outcomes			√		√	
Cardiovascular endpoint						√
Safety observation						
Vital signs		√	√	√	√	
Tests for urine and stool		√			√	
Liver function (ALT, AST)		√			√	
Kidney function (BUN, Cr)		√			√	
ECG		√			√	
Adverse events				√	√	√
Other work						
CRF audit						√

#### Data analysis

SPSS Statistics 20.0 was used for statistical analysis of the data. Measurement data were expressed as mean ± standard deviation (mean ± SD). When comparing the two sets of measurement data, if the normal distribution was in line, the t-test or paired t-test of two independent samples was used. If normal distribution was not present, the Mann–Whitney *U* test, also called the Wilcoxon non-parametric test, was used. The counting data were expressed as frequency or composition ratio and analyzed by Chi-square test. All trial data were double-sided, with a *P* < 0.05 indicating a statistically significant difference.

## Discussion

Coronary heart disease is a major global public health problem, with high morbidity and mortality. Although the use of Western medicine has significantly reduced the mortality rate of patients with coronary heart disease, there are still many patients with chest pain, chest tightness, shortness of breath, fatigue, and other symptoms, resulting in a decline in their quality of life and seriously affecting normal life. Traditional Chinese Medicine takes dialectical treatment as the core in improving symptoms and improving the quality of life, and believes that the pathogenesis of coronary heart disease has kidney deficiency at the foundation, and blood stasis as the target, therefore treatment should be legislated to replenish the kidney and activate blood (10, 11).

BSHXG used in this study is composed of 10 drugs. Modern research has found that Chuanxiong, the main active ingredient of tetramethylpyrazine, can dilate coronary arteries and improve blood oxygen supply to the myocardium (12, 13). The combination of Danshen and Chuanxiong can effectively improve blood rheological indexes including erythrocyte sedimentation rate, fibrinogen, hematocrit, whole blood viscosity, and plasma viscosity in patients with coronary heart disease (14). The total red peony glycosides in Chishao can exert its antithrombotic effect by reducing fibrinogen, red blood cell aggregation, and platelet aggregation (15). At the same time, modern studies have also found that the main component of Roucongrong, Cistanche Total Glycoside, can reduce the amplitude of ST segment elevation of ECG in rats with coronary ligation, reduce the area of myocardial infarction, regulate myocardial phosphocreatine kinase activity, and play a role in protecting ischemic myocardium (16). Bajitian can increase the activity of Ca2+-ATPase and Na+-k+-ATPase enzyme, reduce creatine kinase concentration, myocardial infarction area, and protect cardiomyocytes (17). Gualou has the effects of dilating coronary arteries, anti-platelet aggregation, protecting ischemic myocardium, improving oxygen tolerance, and inhibiting inflammatory reactions (18). Cinnamon in Guizhi has the effect of inhibiting the secretion of prostaglandin E2, and then exerts anti-inflammatory effects (19). *Panax notoginseng* in Sanqi can protect the vascular endothelium (20). Renshen has the effects of anti-atherosclerosis, anti-platelet aggregation, anti-thrombosis, improving myocardial ischemia and ventricular remodeling (21). Goqizi can reduce the content of cholesterol and triglycerides in human serum, reduce the degree of lipid peroxidation, improve superoxide dismutase (SOD) activity, maintain the balance of the body's antioxidant system, protect tissue cells from free radical damage, and reduce blood lipid content (22).
